# The efficacy and safety of Shenfu injection in patients undergoing percutaneous coronary intervention for ST segment elevation myocardial infarction: A protocol for systematic review and meta-analysis

**DOI:** 10.1097/MD.0000000000032483

**Published:** 2023-01-06

**Authors:** Xiafeng Yan, Minya Dong, Xiao Qin, Nannan Li, Na Li

**Affiliations:** a Department of Cardiovascular Medicine, Dongying People’s Hospital, Shandong, 257000China.

**Keywords:** meta-analysis, myocardial infarction, percutaneous coronary intervention, Shenfu injection

## Abstract

Shenfu injection is a traditional Chinese medicine formulation that alleviates ischemia–reperfusion injury through multiple pharmacologic effects. However, there is lack of evidence regarding its efficacy in myocardial infarction. We performed a protocol for systematic review and meta-analysis to evaluate the efficacy and safety of Shenfu injection for ST segment elevation myocardial infarction after primary percutaneous coronary intervention.

**Methods:**

This review has been reported following the preferred reporting items for systematic reviews and meta-analyses protocol. A literature search was performed in November 2022 without restriction to regions, publication types, or languages. The primary sources were the electronic databases of PubMed, Embase, Web of Science, the Cochrane Central Register of Controlled Trials, Chinese National Knowledge Infrastructure database, Chinese Biomedical Database, and Chinese Science and Technology Periodical database. Risk of bias will be assessed according to the Cochrane Risk of Bias Tool. Data analysis was performed using Reviewer Manager 5.4.

**Results:**

The results of this systematic review will be published in a peer-reviewed journal.

**Conclusion:**

This systematic review will provide evidence to judge whether Shenfu injection is effective and safe in patients undergoing percutaneous coronary intervention for ST segment elevation myocardial infarction.

## 1. Introduction

ST segment elevation myocardial infarction (STEMI) remains a major cause of premature death worldwide.^[1,2]^ STEMI accounted for 39% of all hospital admissions due to myocardial infarction in the UK.^[3]^ The unadjusted 30-day mortality rate for STEMI patients was 8.1% during the period 2013 to 2014, compared with 12.4% ten years earlier^[4]^; such an impressive decline can be attributed to improvements in emergency medical response, adoption of effective reperfusion strategies, and widespread use of secondary preventive pharmacotherapy.^[5]^

Despite recent advances, controversies persist regarding its optimal management. Most STEMI are caused by atherosclerotic plaque rupture with vessel occlusion due to secondary thrombosis, with the extent of subsequent myocardial injury dependent on the area of myocardium subtended by the culprit vessel, duration of occlusion and presence of collaterals.^[6,7]^ Primary percutaneous coronary intervention (PCI) is the preferred treatment in patients with STEMI.^[8,9]^ Although there have been large improvements in the outcomes for these patients, subgroups at high risk for mortality remain.

Shenfu injection is a traditional Chinese medicine formulation containing ginseng (Panax; family: Araliaceae) and aconite (Radix aconiti lateralis preparata, Aconitum carmichaeli Debx; family: Ranunculaceae), with ginsenosides and aconite alkaloids as the main active ingredients.^[10,11]^ Animal studies have shown that Shenfu injection has protective effects against reperfusion injury through multiple pharmacologic effects, such as scavenging free radicals, inhibiting inflammatory mediators, suppressing cell apoptosis, and inhibiting calcium overload.^[12]^ However, no data are available regarding its efficacy in patients undergoing PCI for STEMI. In addition, the mechanism of Shenfu injection for treating STEMI was unclear.

This present study is performed to verify the efficacy and safety of Shenfu injection for STEMI after primary PCI with clinical evidence-based studies, and systematically elaborate the specific role of Shenfu injection for these patients, which will be served as the guidance for a multicenter random control trial in our further research.

## 2. Methods

### 2.1. Study registration

This review protocol is registered in the PROSPERO International Prospective Register of systematic reviews, registration number CRD42022372522. It has been reported following the preferred reporting items for systematic reviews and meta-analyses protocol.^[13]^ All studies included in this meta-analysis come from public research databases. Ethical approval is not required for this study.

### 2.2. Inclusion criteria for study selection according to PICOS criteria

Participants: Adult patients more than 18 years old with clinically diagnosis of STEMI undergoing PCI will be included. Any patient with cardiac surgery, other serious complications, or cardiac assistive equipment like implantable cardioverter defibrillator will be excluded. Interventions: the intervention of the experimental group received intravenous Shenfu injection. Comparison: control group received same amount of normal saline or matched placebo. Outcomes measures: the major outcomes included mortality, clinical total effective; secondary outcomes included the left ventricular ejection fractions and 6-minute walk test. Study design: Only randomized controlled trials will be included. Other types of researches like observational studies will be excluded.

### 2.3. Search strategy

A literature search was performed in November 2022 without restriction to regions, publication types or languages. The primary sources were the electronic databases of PubMed, Embase, Web of Science, the Cochrane Central Register of Controlled Trials, Chinese National Knowledge Infrastructure database, Chinese Biomedical Database, and Chinese Science and Technology Periodical database. Two authors will independently draft and carry out the search strategy. The gray literature will be searched in databases. Articles will also be searched from the references of the retrieved studies. The key terms used for the search are “Shenfu injection,” “percutaneous coronary intervention,” and “myocardial infarction.” The search strategy used in PubMed is presented in Table 1.

**Table 1:** Search strategy (PubMed).

Number Search terms
#1 myocardial infarction [Ti/Ab]
#2 acute coronary syndrome [Ti/Ab]
#3 coronary heart disease [Ti/Ab]
#4 AMI [Ti/Ab]
#5 STEMI [Ti/Ab]
#6 coronary infarction [Ti/Ab]
#7 coronary atherosclerotic heart disease [Ti/Ab]
#8 #1 OR #2 OR #3 OR #4 OR #5 OR #6 OR #7
#9 percutaneous coronary intervention [Ti/Ab]
#10 reperfusion therapy [Ti/Ab]
#11 stent [Ti/Ab]
#12 interventional therapy [Ti/Ab]
#13 #9 OR #10 OR #11 OR #12
#14 shenfu injection [Ti/Ab]
#15 ginseng [Ti/Ab]
#16 Chinese herbal medicine [Ti/Ab]
#17 #14 OR #15 OR #16
#18 #8 AND #13 AND #17

### 2.4. Study selection

**Figure 1.**
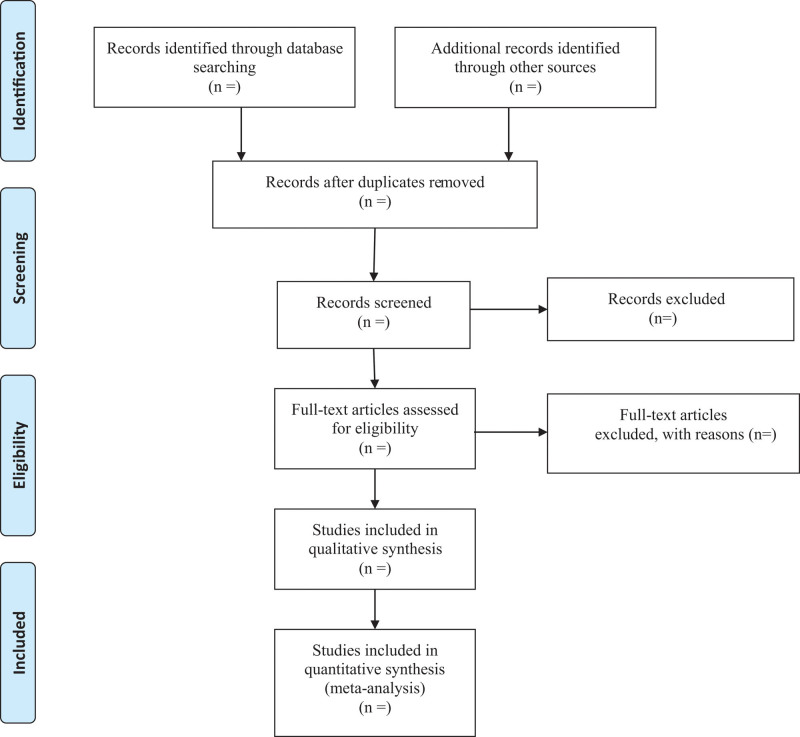
Flow diagram.

Study selection will be carried out by 2 independent experienced researchers. Endnote X9 acts as a literature management tool for retrieved articles. After the software automatically removes duplicate references, they will screen preliminarily the title and abstract to determine whether inclusion criteria are met. Subsequently the selected literature will be downloaded in full text for more detailed screening. In case of controversy, a third investigator will join the discussion. All of the excluded articles will be marked with reasons. The whole selection process will be presented in a PRISMA flow diagram (Fig. 1).

### 2.5. Data extraction

The following information will be collected by a predetermined data form generated by Microsoft Excel: basic information (title, first author, year of publication, type of study, location of study); study population (age, sex, sample size, detailed description of participants, diseases); details of interventions and comparison; related outcomes mentioned above and the length of follow-up time. Any disagreement in this process will be solved by consensus or consultation with a third person.

### 2.6. Risk of bias

Risk of bias will be assessed according to the Cochrane Risk of Bias Tool^[14]^ which bases on the following domains: random sequence generation, allocation concealment, blinding of participants and personnel, blinding of outcome assessment, incomplete outcome data, selective outcome reporting, and other sources of bias. Items were scored as low, high, or unclear risk of bias. Two independent researchers will attend the evaluation which will be cross-checked by a third senior one.

### 2.7. Data synthesis

The meta-analysis was performed using Reviewer Manager 5.4. The 95% confidence intervals were used, mean differences were calculated for continuous variables, and risk ratios were calculated for dichotomous variables. Data heterogeneity was assessed using the chi-square and *I*^2^ tests. When heterogeneity was not significant (*P* ≥ .1, *I*^2^ ≤ 50%), a fixed-effects model was used for analysis; when heterogeneity was significant (*I*^2^ > 50% or *P* < .1), a random-effects model was used. In cases of significant heterogeneity among the included studies, we performed a subgroup analysis. This will be explored according to age, gender, race, treatment period, sample size, disease category, and other factors that may affect the results.

## 3. Discussion

The introduction of primary PCI for STEMI allows timely reperfusion for limiting infarct size and subsequent cardiac remodeling.^[7,15]^ However, reperfusion can trigger irreversible myocardial injury, a phenomenon called reperfusion injury. Many cardioprotective therapies have been proposed to reduce infarct size but have shown inconsistent clinical efficacy. As a Chinese herbal formula, Shenfu injection is characterized by multicomponents and multiple pharmacologic effects. Ginseng and aconite, the main components of Shenfu injection, have the effect of restoring *yang* and saving *adversity*, replenishing *qi*, and relieving detoxification, which has been significantly effective in the treatment of coronary heart disease, and its protective effect on heart has been proved.^[16]^ Ginsenosides, the main active ingredient in ginseng, account for 4% of the total ginseng, which can improve the immunity of the body^[17]^; the active ingredients of aconite have cardiotonic and antiarrhythmic effects.^[18]^ This is the first meta-analysis to evaluate the efficacy and safety of Shenfu injection for STEMI after primary PCI. The results of our study need to be validated in larger trials (particularly confining to patients with estimated larger infarct size).

## Author contributions

**Data curation:** Nannan Li.

**Methodology:** Xiao Qin.

**Validation:** Minya Dong.

**Writing—original draft:** Xiafeng Yan.

**Writing—review and editing:** Na Li.
